# Somatic evolutionary timings of driver mutations

**DOI:** 10.1186/s12885-017-3977-y

**Published:** 2018-01-18

**Authors:** Karen Gomez, Sayaka Miura, Louise A. Huuki, Brianna S. Spell, Jeffrey P. Townsend, Sudhir Kumar

**Affiliations:** 1 Institute for Genomics and Evolutionary Medicine, Sudhir Kumar, SERC 602A, 1925 N. 12th Street, Philadelphia, PA 19122 USA; 2 Department of Biology, Temple University, Philadelphia, PA 19122 USA; 3 Department of Biostatistics, Yale School of Public Health, New Haven, Connecticut 06510 USA; 4 Department of Ecology and Evolutionary Biology, Yale University, New Haven, Connecticut 06511 USA; 5 Program in Computational Biology and Bioinformatics, Yale University, New Haven, Connecticut 06511 USA; 6 Center for Genomic Medicine and Research, King Abdulaziz University, Jeddah, Saudi Arabia

**Keywords:** Driver mutation, Ubiquitous mutation, Private mutation, Somatic mutation

## Abstract

**Background:**

A unified analysis of DNA sequences from hundreds of tumors concluded that the driver mutations primarily occur in the earliest stages of cancer formation, with relatively few driver mutation events detected in the late-arising subclones. However, emerging evidence from the sequencing of multiple tumors and tumor regions per individual suggests that late-arising subclones with additional driver mutations are underestimated in single-sample analyses.

**Methods:**

To test whether driver mutations generally map to early tumor development, we examined multi-regional tumor sequencing data from 101 individuals reported in 11 published studies. Following previous studies, we annotated mutations as early-arising when all tumors/regions had those mutations (ubiquitous). We then inferred the fraction of mutations occurring early and compared it with late-arising mutations that were found in only single tumors/regions.

**Results:**

While a large fraction of driver mutations in tumors occurred relatively early in cancers, later driver mutations occurred at least as frequently as the early drivers in a substantial number of patients. This result was robust to many different approaches to annotate driver mutations. The relative frequency of early and late driver mutations varied among patients of the same cancer type and in different cancer types. We found that previous reports of the preponderance of early driver mutations were primarily informed by analysis of single tumor variant allele profiles, with which it is challenging to clearly distinguish between early and late drivers.

**Conclusions:**

The origin and preponderance of new driver mutations are not limited to early stages of tumor evolution, with different tumors and regions showing distinct driver mutations and, consequently, distinct characteristics. Therefore, tumors with extensive intratumor heterogeneity appear to have many newly acquired drivers.

**Electronic supplementary material:**

The online version of this article (doi: 10.1186/s12885-017-3977-y) contains supplementary material, which is available to authorized users.

## Background

Tumor cells accumulate numerous somatic mutations. Some of these mutations directly contribute to tumor growth and progression and are commonly referred to as driver mutations. Knowledge of the relative timing of driver mutations is essential for understanding cancer progression as a whole and for optimizing treatment for individual patients [1–4]. For this reason, much attention has been paid to identifying the distribution and frequency of driver mutations among tumor cell populations [5–7].

Mutations found in most cells of a tumor can be detected by estimating the fraction of cancer cells harboring a particular variant, or cancer cell fraction (CCF) [8]. This approach has been utilized to analyze the extensive data available through the Cancer Genome Atlas (TCGA), a comprehensive database that contains genomic changes in hundreds of thousands of tumors (single tumor genome sequencing results) from 33 types of cancers [9]. When CCFs were estimated for driver mutations found in tumors from TCGA, most driver mutations were present at high CCF, meaning most driver mutations were found in the majority of cells in a tumor [10]. Mutations found in a large proportion of tumor cells are likely to have occurred at the earlier stages of tumor growth, because such early-arising mutations are inherited by all cells in a tumor following clonal evolution [2, 11]. Therefore, it was inferred that the majority (>70%) of driver mutations occur early in tumor growth for all types of cancer surveyed [12].

However, tumors consist of heterogeneous cell lineages, each of which may be driven by different driver mutations [1–3]. For example, a study of renal cell carcinoma reported 73–75% of driver mutations to have likely occurred at a later time in tumor evolution [13]. This study employed a different methodology to detect early mutations, where variants from multiple regions of a given tumor (M-seq) were analyzed. Using an M-seq approach, variants found in a majority of sampled regions are classified as early (ubiquitous) mutations [6]. Additional studies have employed M-seq methodology to study different types of cancer, and these studies have identified many mutations that are private to one or only a few regions of a tumor [14–21], or mutations that are present at different points in time [22, 23]. This identification of many private mutations indicates that single-tumor profiles, such as those in the TCGA database, do not completely capture the spectrum of late-arising driver mutations. Therefore, conclusions based on analyses of TCGA data [12] may not apply to all the cancers and/or patients.

Now that M-seq data from various cancers are available, it is possible to comprehensively explore the relative preponderance of driver mutations arising in early and late stages of tumor growth. Here we perform a meta-analysis using samples from 101 individuals representing various cancer types [13, 14, 24–32], which revealed that the fraction of driver mutations occurring early in tumor growth varies extensively among cancers as well as among individuals. We evaluate the frequency of late-arising driver mutations in primary and metastatic tumors.

## Methods

We obtained sequencing read counts of mutant and wild type alleles, and their chromosomal positions, for 101 tumor data sets from 11 published studies that contained at least three tumor samples per patient (Table 1) [13, 14, 24–32]. Our analysis focused on single nucleotide variants (SNVs) and insertions and deletions (indels) that arose somatically in the tumors of individual patients. To identify mutations that likely affected cancer progression or development (driver mutations), we first extracted mutations in coding regions of the genome by mapping the chromosomal position of each variant onto the reference human genome (hg19) from the Ensembl database [33]. We excluded mutations in intergenic regions, because their functional effects on cancer development are rarely known. We also excluded synonymous mutations, because these mutations are not expected to significantly affect protein function.

**Table 1:** Summary of data sources analyzed in the present study

Cancer type	Study (Reference)	Number of Patients in Study
Mixed: Lung, pancreatic, and 11 other types	[32]	37
Brain (glioblastoma)	[25]	10
Colorectal	[27]	9
Prostate	[31]	9
Kidney (clear cell renal cell carcinoma)	[13]	8
Lung	[14]	7
Endometrial	[28]	6
Esophageal (with Barrett’s esophagus)	[29]	5
Brain (glioma)	[24]	5
Breast	[26]	4
Ovarian	[30]	1

To ensure that our findings regarding the distribution of drivers are robust to methods of driver determination, we used five schemes to determine mutations that are cancer drivers. We first determined driver mutations based on whether the affected gene has been previously implicated in cancer. We annotated every mutation occurring in a cancer-associated gene listed in the COSMIC cancer gene census [34, 35] as a driver (Driver annotation type I). Among these genes, those without functional annotation in cancer (oncogene or tumor suppressor gene) could be false-positives. Therefore, we applied a second, more stringent approach, in which we used only known oncogenes and tumor suppressor genes listed in COSMIC (Driver annotation type II).

Some sites in the genome are more frequently mutated in cancer than others, and these somatic variant hot spots are believed to play a role in cancer [36]. In our third approach, we annotated driver mutations when mutations were located within 15 nucleotides of somatic variant hot spot (Driver annotation type III), because mutations at hot spots and neighboring regions may be cancer drivers. Variant hot spots were those identified as individual substitution hot spots as presented in Chang et al. [36], those mutated >10 individuals in the COSMIC database, or those mutated in at least two individuals in our 101 datasets.

Furthermore, many computational methods have been created to determine variants with functional roles in cancer [37]. In the fourth approach, we used one such driver mutation prediction tool, IntOGen [38] (http://www.intogen.org/analysis). The IntOGen pipeline examines genes that frequently have mutations with high functional impact and regions of the protein sequences where mutations frequently occur (Driver annotation type IV). To predict driver mutations with IntOGen, we input all mutations (point mutations and indels) with chromosomal positions, wild type and mutant nucleotides, and strand information obtained from Ensembl database.

Lastly, it has been observed that genes that are causal in one cancer type many not be causal in every cancer type. Therefore, in annotation type V, we obtained a list of cancer associated genes for each cancer type from IntoGen database [38] in order to designate driver mutations following the approach outlined in annotation type I above. For analysis of data from Zhao et al. [32], we used cancer-associated genes identified for each patient by Zhao et al. to maintain consistency with their analysis.

Before distinguishing between early vs. late-occurring mutations, we made use of the extra power conferred by the M-seq approach to detect potential sequencing errors. Using Treeomics [39], we estimated the posterior probability that a putative variant read is actually present in a tumor given the reference and mutant allele read counts for multiple tumor samples. For each variant within a tumor sector, we annotated a mutation as present if the Treeomics-inferred posterior probability was greater than 0.95. We removed variants with a posterior probability less than 0.95 in all tumor samples from a patient. Similarly, a mutation was annotated to be absent if the Treeomics-inferred posterior probability was less than 0.05.

To distinguish early from late-arising mutations, we defined early-arising mutations as those that are found in all samples (ubiquitous mutations), and late-arising mutations as those that are private to only one sample (region-specific or private mutations). This definition is more stringent than the definition used in the previous TCGA analysis [12], because it excludes mutations found in some, but not all tumors. Consequently, mutations annotated to be early-arising are expected to be found in the progenitor of all tumor cells in a patient, and those designated late-arising status are expected to have arisen in only one tumor in a patient. We refer to all other drivers to be of intermediate origin. Note that early and late designations refer to relative timing of occurrence of mutations, they are not meant to convey absolute times. Supplementary Additional file 1 shows designations of drivers using five annotation schemes, chromosomal positions, count of samples with mutant allele after Treeomics treatment, patient ID, and study information.

## Results

### Meta-analysis of driver mutation timing

**Fig. 1:**
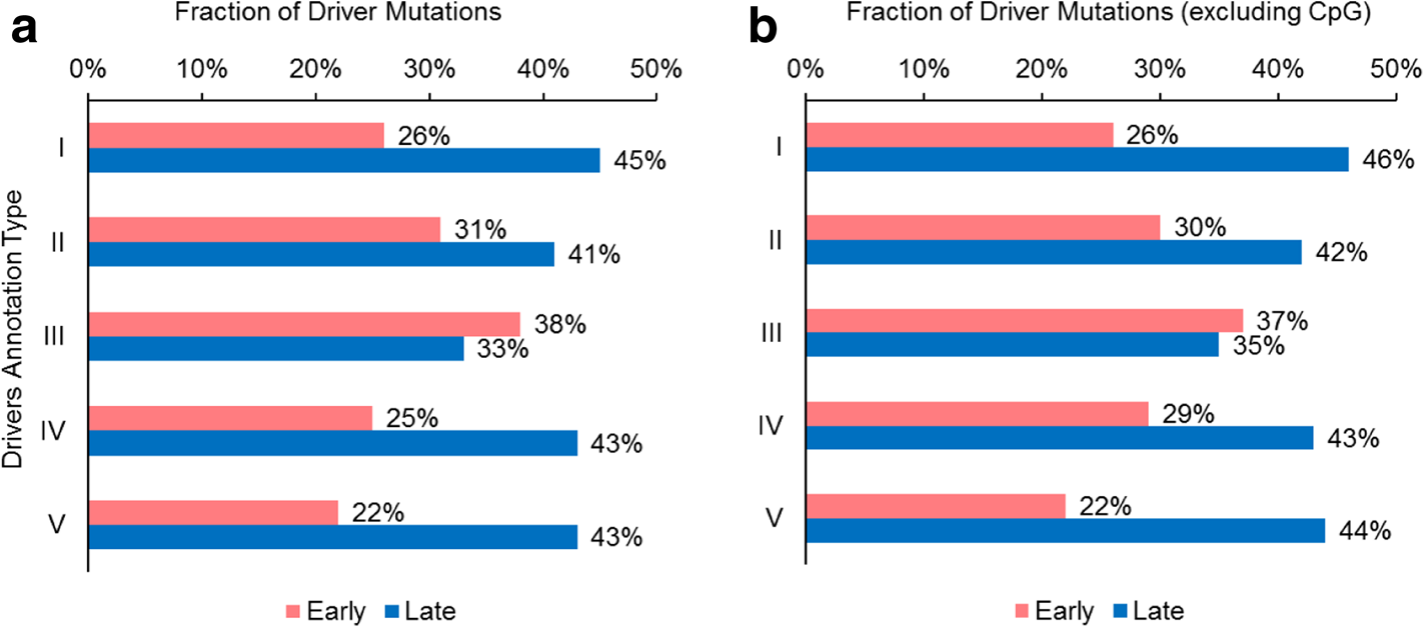
Overall timing of driver mutations**.** The fraction of driver mutations that are early (pink) and late (blue) are shown for each of the driver mutation annotation schemes (I–V, see **Methods**), **a**) including CpG sites, and **b**) after the removal of CpG sites

We first pooled driver mutations from 101 patient data sets from 11 studies [13, 14, 24–32] and identified early drivers. Analyzing type I drivers (i.e., any mutation in a cancer-associated gene [34, 35]), we found that only 26% of all driver gene mutations have arisen early (Fig. 1a), which indicates that most driver mutations did not occur at the earliest stages of tumor growth. In fact, we found that 74% of driver mutations were not early, which is in contrast to results from TCGA analysis [12], but consistent with the findings of Gerlinger et al. [13] in clear cell renal carcinoma samples. However, our data sample is 15 times larger than Gerlinger et al. [13] and includes tumors from 21 types of cancers. These findings were robust to the use of more stringent approaches to driver gene determination (annotation type II to V): the number of non-early driver mutations was always greater than the number of early driver mutations. Across annotation types, only about one-third of all driver mutations occurred early.

Interestingly, we found relatively large numbers of driver mutations to be late-occurring (33–45%), with the number of late-driver mutations similar to or even greater than the number of early-occurring driver mutations (22–38%) (Fig. 1a). The remaining driver mutations had intermediate origins. In these analyses, only annotation type III predicted early driver mutations (38%) to be larger than late driver mutations (33%), but still they are very similar in numbers. That is, across M-seq cases, late-occurring drivers are generally more frequently observed than, or are equal in frequency to, early-arising drivers. Analysis using the less stringent definition of driver mutation (annotation type I) produced results similar to the more stringent definitions (annotation types II–V). Overall, we found the numbers of late-arising driver mutations to be substantial.

False-positive detections of driver mutations are expected to be high when mutations are located on genomic positions with higher mutation rate, e.g., CpG sites [40, 41]. Therefore, we obtained a list of CpG sites in the human genome from UCSC sequence hg19 using the Bioconductor R package, and removed driver mutations that were located at these sites. We still observed fewer driver mutations to have occurred early (22–37%) than late (35–46%; Fig. 1b). So, we expect that our inferences have not been affected by false-positive detection of driver mutations at mutational hot spots.

### Driver mutation timing by cancer type and individual differences

**Fig. 2:**
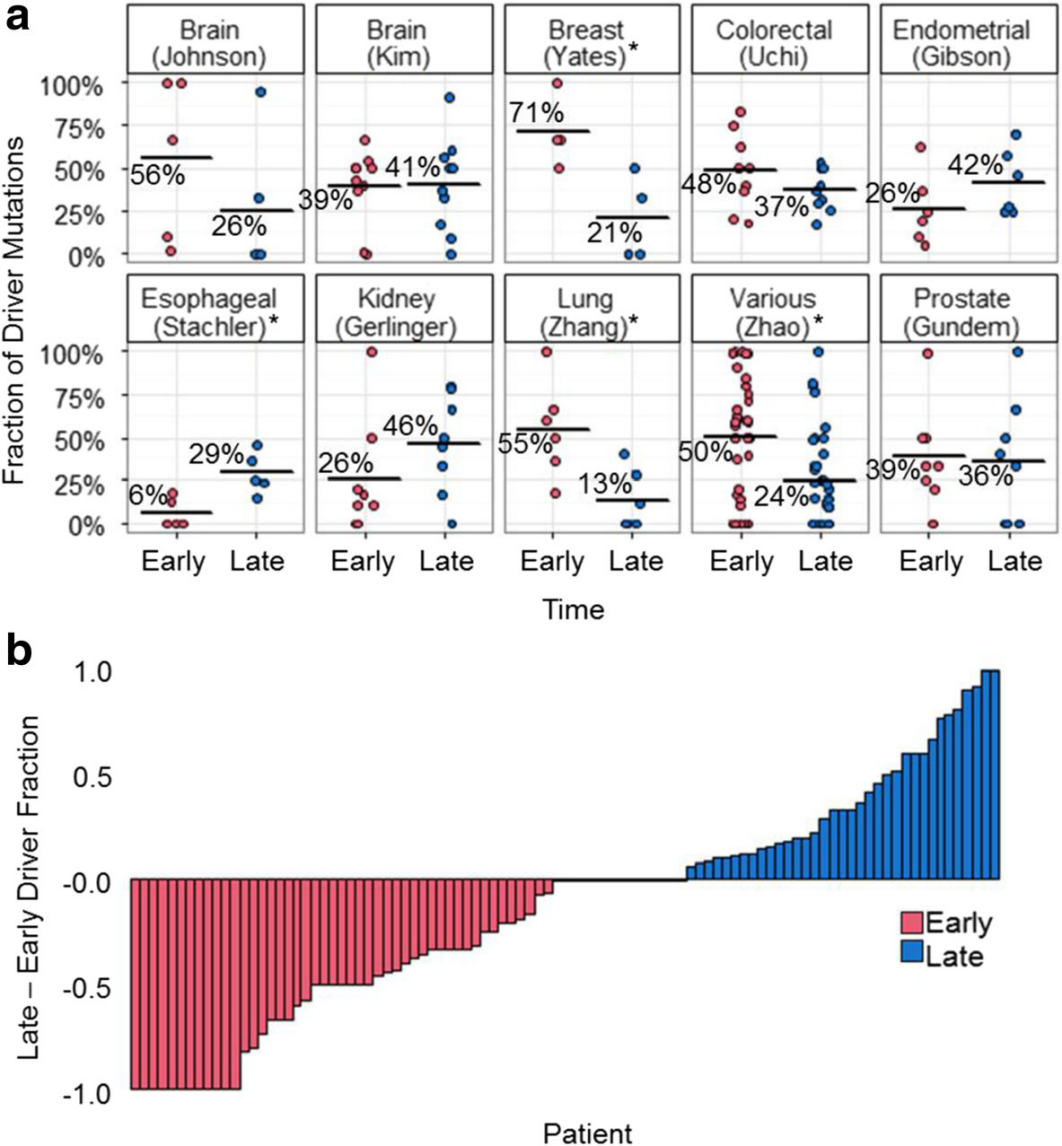
Fraction of driver mutations occurring at early and late time. Driver mutations were annotated as those found in cancer-associated genes. **a** Fraction of all driver mutations that occurred early and late as inferred from multi-sample profiles. Each dot refers to data from one patient from a study, and a bar shows the average. Statistical tests (paired *t*-test) were performed to test if the fraction of early-driver mutations is significantly different from late-driver mutations for a cancer type. Cancer types that have significantly different fractions (*P* ≤ 0.05) are shown with asterisks. **b** The difference in early and late driver mutation fractions for individual patients. Zero difference was found for 15 patients. Three data sets were removed because there were zero driver mutations after removing variants absent from all tumors after the application of Treeomics software (see **Methods**)

Although the total number of late driver mutations in our data was similar to or greater than that of early driver mutations, we found that the fraction of driver mutations occurring at early stages varied extensively among patients, studies, and cancer types. Very few early drivers were detected in esophageal adenocarcinoma data sets (average 6%, range 0–18%), but a large fraction of drivers were early in breast cancer data sets (average 69%, range 50–100%; Fig. 2a). Similarly, the average fraction of late mutations had a wide range, from 13% in ovarian cancer to 81% (range 0–91%) in recurrent glioblastoma.

Although patients showed similar fractions of early and late driver mutations within specific cancer types, some cancer types did not. For example, there is extensive variation in the fraction of early and late mutations identified in both glioblastoma data sets sequenced at primary tumor and recurrence [24, 25]. Similarly, analyses of primary tumor and multiple metastatic tumors for each patient (Zhao’s [32] data) revealed extensive variation among patients in the fraction of early and late mutations. About half of the patients (48 patients) exhibited a larger fraction of early driver mutations than late driver mutations (Fig. 2b). Furthermore, we found that a large number of patients (35 patients) exhibited a greater fraction of late driver mutations than early driver mutations. Therefore, the relative counts of early to late driver mutations in a tumor varied both by tumor type as well as on an individual basis.

**Fig. 3:**
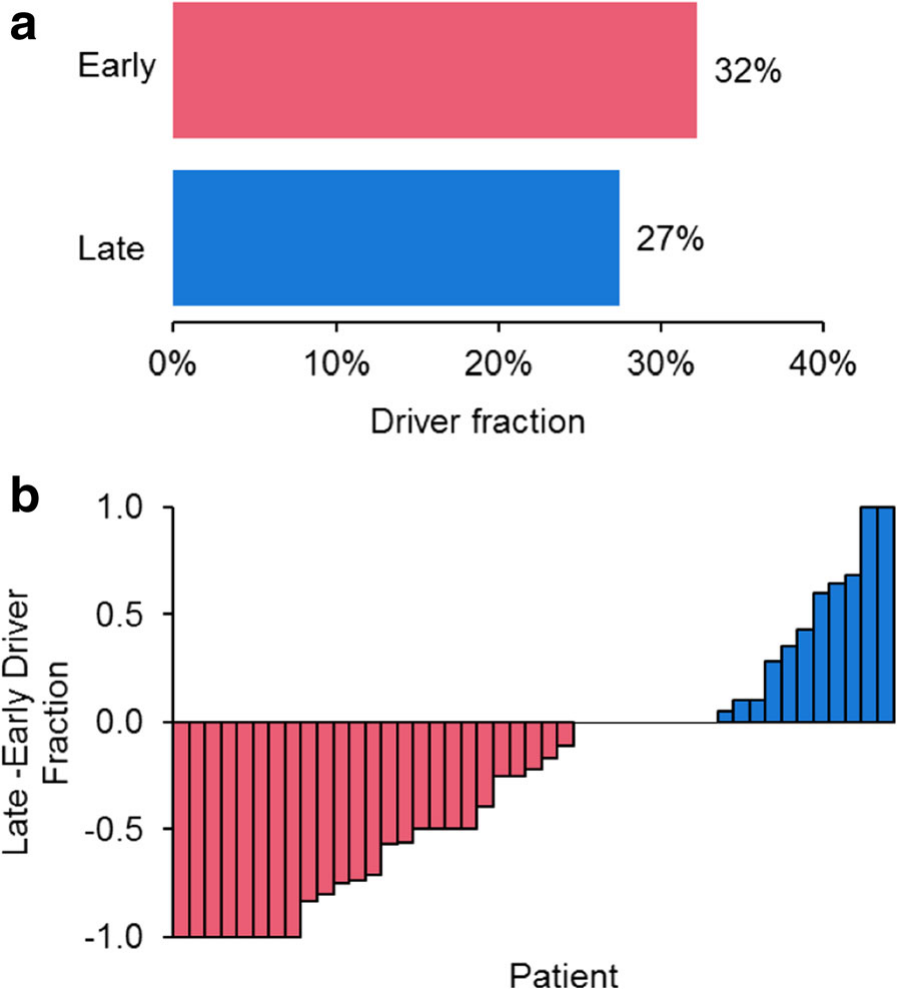
Fraction of early and late driver mutations in metastatic tumors. **a** The fraction of driver mutations that are early and late. **b** Difference between late-and early-driver mutation fraction. Each bar represents a patient: pink marks patients that have a greater fraction of early-driver mutations than late, and blue marks patients that show an opposite trend. Nine patients showed zero difference

We also analyzed the preponderance of late drivers found in metastatic tumors, because mutations found in metastatic tumors can be classified as occurring late with greater certainty than those mutations found in the primary tumors as well. We found that the fraction of early driver mutations detected in this way remained similar to those reported above (32%), and late drivers still occurred at a high frequency (27%; Fig. 3a). That is, the fraction of late driver mutations was only slightly smaller than early driver mutations (27 and 32%), with some patients showing larger numbers of late mutations than early mutations (Fig. 3b).

### Single versus multiple tumor profiles

**Fig. 4:**
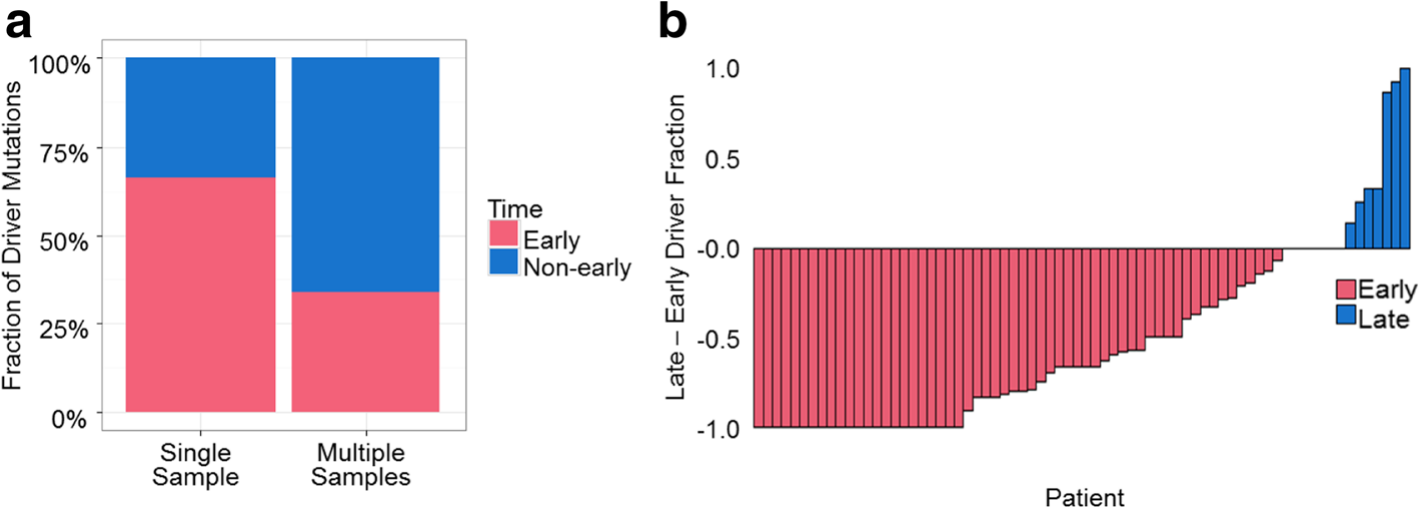
Timing of driver mutations using single and multiple tumor samples. Driver mutations were annotated as those found in driver genes identified in the previous report [12]. **a** Fraction of driver mutations occurring at early time. For the single sample data set (left), we generated 100 replicates, where we randomly selected a single sector per patient. For each replicate, we pooled driver mutations and computed the fraction of early driver mutations (mean: 66%). For multiple samples (right), all samples available for each data set were used to compute the fraction of early driver mutations (45%). The fraction of early drivers found in 100 replicates of single-tumor sampling was statistically greater than the early driver fraction found using multiple samples by single single-sample t test (*P* < 10^−15^). **b** Difference between late-and early-driver mutation fraction calculated using single-tumor samples (one replicate is shown). Each bar represents a patient: pink marks patients that have a greater fraction of early-driver mutations than late, and blue marks patients that show an opposite trend. Eleven patients contained equal proportions of early and late drivers, and 7 patients were removed as no driver mutations were identified

We tested the hypothesis that the use of only a single tumor per patient in previous analyses is the primary reason for the difference between our results and those reported earlier (e.g., [12]). Using just one tumor sample for each patient in our datasets and applying the driver annotation scheme as in McGranahan et al. [12], we found that 66% of the drivers were inferred to be early, which is consistent with McGranahan et al. [12]’s finding of 70% or more of drivers originating early-on (Fig. 4a). This fraction decreased dramatically (to 45%) when multiple samples are used for each patient from the same data set. Therefore, the power to detect late driver mutations is strongly dependent on the use of multiple samples per patient.

In addition, very few patients had a greater fraction of late-drivers compared to early-drivers when single samples were used (Fig. 4b). This comparison reveals that the use of a single sample per patient leads to a different result from that obtained using multiple samples, and the power to detect late-occurring drivers increases with additional sampling. This result is consistent with those reported previously [6, 32, 42]: multiple sequenced regions are necessary to determine the numbers of early and late driver mutations [43]. Overall, the use of single tumor samples provides poor scope to differentiate driver mutation events that happen early in tumor growth from late-arising driver mutations.

### Robustness of early vs. late driver occurrence patterns

While the above patterns consistently showed that the numbers of driver mutations occurring late are comparable to those that occurred early-on, it is important to assess their robustness to a number of factors that complicate analysis and interpretation of tumor genome variation.

First, the observed variability in the timing of driver mutation occurrence among patients may be caused by technical issues, such as mutation calling methods, tumor purity, and sequencing depth. This was the reason for our use of Treeomics to exclude low quality SNVs due to low sequencing depth.

Second, it is possible that the differences observed between studies (cancer types) were caused by the differences in mutation calling methods among the studies, as some studies may be able to detect mutations with lower SNV frequencies than others. However, we often observed that the fraction of early driver mutations as well as late driver mutations varied among patients from the same study analyzed with the same methodologies. Therefore, any systematic error based on methodology would appear to be minor, and such technical issues should not strongly affect our conclusion.

Third, tumor purity could impact the annotation of early and late drivers. Generally, though not necessarily, the late-arising subclones will be in lower frequency when the tumor purity is low. Therefore, if purity were an issue, we would expect to experience a lesser power to detect late drivers as compared to early drivers, as early drivers are expected to manifest at higher frequencies. Thus, our estimates of the relative excess of late drivers are likely to be conservative.

Fourth, the number of early driver mutations may be underestimated, because sequencing reads indicating true early driver mutations may not be observed in one of many samples by chance, despite high overall coverages. While this dropout can occur, we expect it to be far less common for early drivers than it would be for private mutations, because they will generally occur with lower frequency and in fewer multiregion samples than the early drivers. Once again, our observation of the relative excess of late drivers is conservative.

**Fig. 5:**
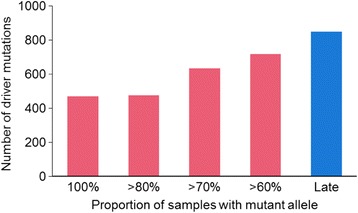
The number of early driver mutations when some samples may have wild-type alleles. We annotated mutations as early mutations, when 100% (all), >80, >70, and >60% of samples had mutant alleles. The number of late driver mutations are shown with the blue bar

Fifth, copy number alternations (CNAs) will likely cause difficulty in designating some early-arising drivers, because the drivers can be lost by the loss of genomic segments in some tumor samples. Ideally, a reanalysis of all the primary data will be desired to identify this effect fully. However, currently available methods are only modestly accurate [44, 45]. Furthermore, CNAs can occur multiple times during the clonal evolution, which will result in complex evolutionary trajectories for SNVs involved in CNAs. In general, we expect our results to be not severely impacted by CNAs, because the number of SNVs affected by loss of mutant alleles due to CNAs is expected to be small due to the fact that most of CNAs will not affect the presence of mutant alleles, i.e., mutant alleles will be lost only when segmental losses or losses of heterozygosity (LOHs) lead to the loss of mutant alleles. To examine the potential effect of CNAs on the counts of early driver mutations, we annotated mutations as ‘early,’ when >80, >70, and >60% of samples had mutant alleles. Although the number of early driver mutations was increased as we used a less stringent criterion (i.e., allowing some samples without mutant alleles), the number did not exceed the number of late driver mutations (Fig. 5). Therefore, our conclusion should be robust to CNAs.

## Discussion

Our results establish that the fraction of driver mutations occurring in the earliest stages of cancer varies among patients as well as cancer types. We have shown that, overall, the number of late driver mutations are equal to or greater than early drivers in 44% of the patients with metastatic tumors. This conclusion differs from some previous reports arguing that the majority of driver mutations happen early in cancer progression [12] or that tumors follow a neutral pattern of evolution after initial growth propelled by the effects of early driver mutations, i.e., intratumor heterogeneity is caused by passenger mutations [46].

**Fig. 6:**
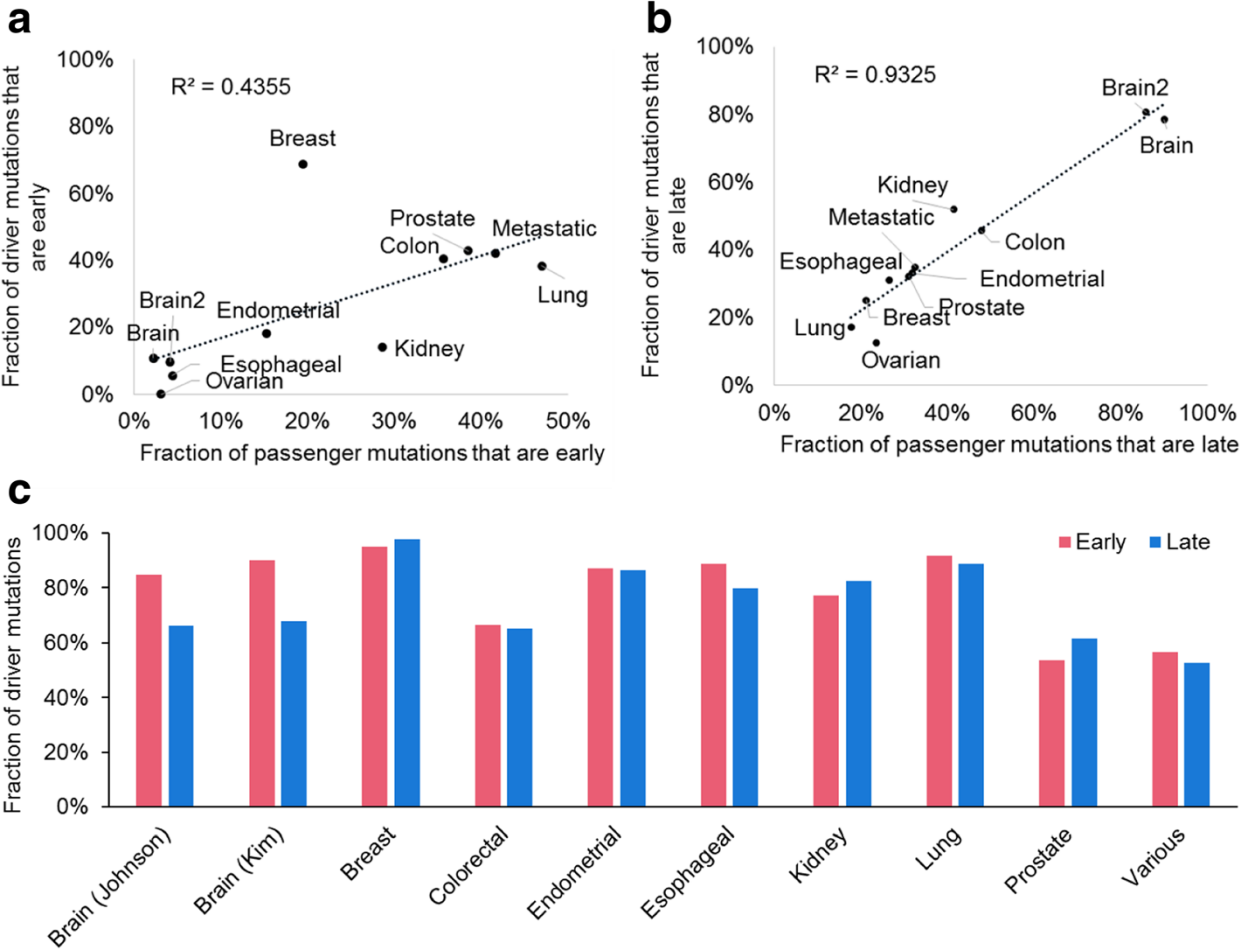
Numbers of driver mutations and passenger mutations. The number of mutations were pooled for each study. **a** and **b** The fractions of driver and passenger mutations that are (**a**) early and (**b**) late. **c** The fractions of driver mutations over total mutations (driver and passenger mutations) for early (pink) and late (blue)

Our observation that the number of late driver mutations is similar to early driver mutations does not inform us about the rate of driver mutation occurrence per cell or about the relative degrees of selective advantage conferred by early and late drivers. The number of cells that arise late in cancer progression (subclonal cells that have subclonal variants) is expected to exceed the number of clonal cells, so the number of subclonal variants is expected to exceed the number of clonal variants, which would result in the increased preponderance of mutations. In fact, the number of driver mutations was linearly correlated with the number of passenger mutations for both early (Fig. 6a) and for late (Fig. 6b) mutations (see also [32]). Actually, the number of early driver mutations per all early mutations (fraction of driver mutations) was similar to the fraction of late driver mutations (Fig. 6c). This pattern was different from the fractions of early and late driver mutations (Fig. 1). Thus, even when the numbers of early and late driver mutations are similar, it will not mean that the rate of driver mutation occurrence or accumulation per cell is the same.

**Fig. 7:**
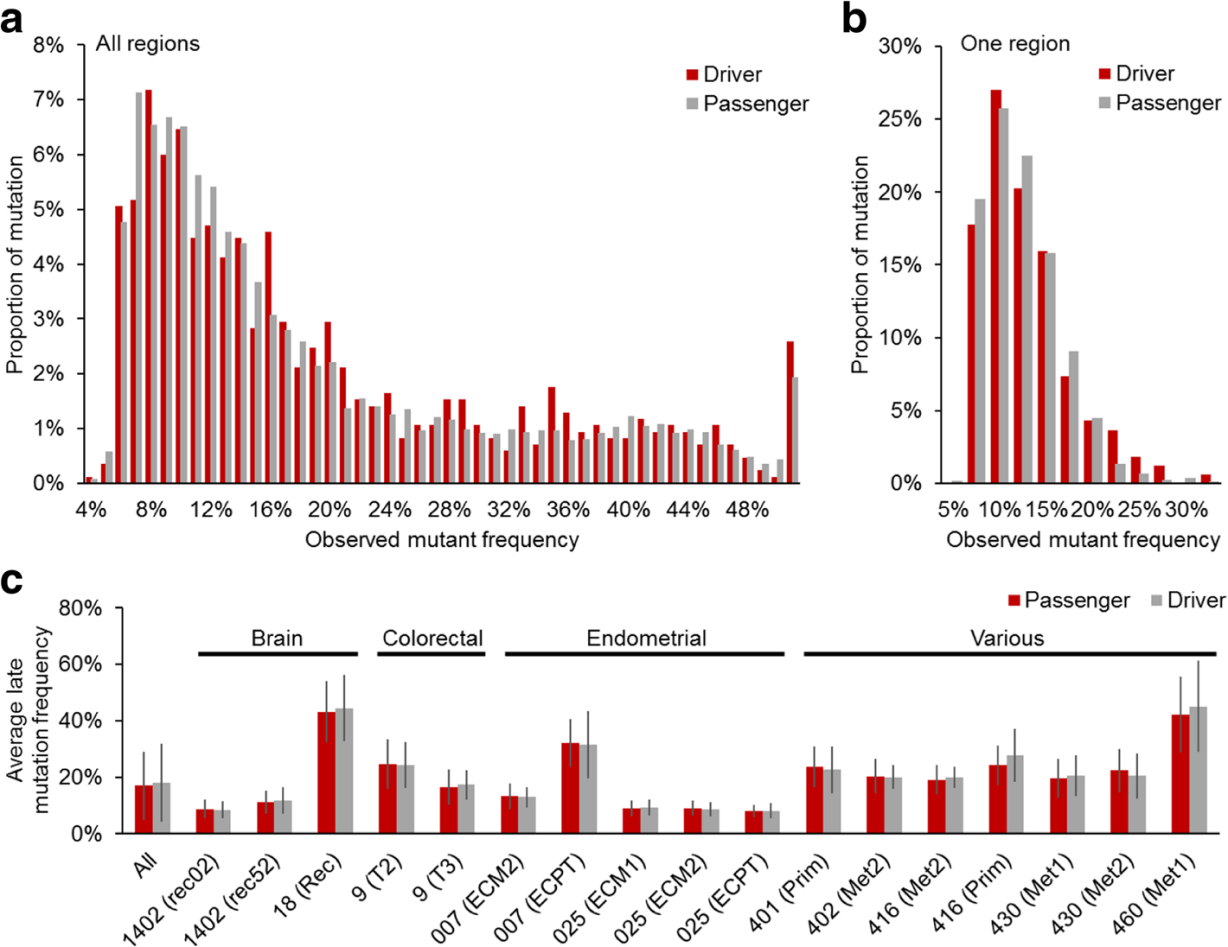
Observed mutant frequencies of late mutations. Observed mutant frequencies were computed by dividing the number of mutant read counts by the number of total read counts. **a** Mutaant frequency distribution where all late mutations were pooled together. **b** Histogram for one region with the largest number of late driver mutations (163 mutations). The data are from region rec52 from the patient 1402 [25]. **c** Regional average mutant frequencies of late drivers and late passengers for all Regions with at least 10 late driver mutations. Patient IDs are presented along x-axis, and region IDs are shown within parentheses. The differences of mutant frequencies between driver and passenger were not statistically significant in any region (*P* > 0.05; *t*-test). Also, the results of all late mutations pooled from all regions are shown (All; *P* = 0.01 by *t*-test, while the difference was only 1%). Error bars are standard errors. Driver and passenger mutations are shown with red and gray bars, respectively

We found that subclones with late drivers occur with significant frequencies; the average observed mutant frequency of late-arising mutations was 19% (with a standard deviation of 14%). In fact, 34% of late mutations were present at frequencies greater than 20% (Fig. 7a). However, the relative degrees of selective advantage conferred by early and late drivers is complex to assess from such frequency data, as for example, subclonal expansions may be caused by spatial constrain without positive selection [46]. Furthermore, a comparison of the frequency of late driver and passenger mutations is not able to inform about positive selection, because passenger mutations hitchhike with driver mutations—which would result in similar observed mutant frequencies for both [47]. As expected, the distribution of the observed mutant frequencies of late drivers was similar to that of late passengers. This pattern was also observed when all the data from all late mutations was pooled together (Fig. 7a) and when the comparison was restricted to individual regions that contained at least 10 late driver mutations (>10 mutations; Fig. 7b, c). However, it does appear that higher intratumor heterogeneity in the late stages is a result of the continued occurrence of genuine driver mutations with functional effects on tumor growth, because recent studies have found subclone-specific driver mutations in tumors using single-cell sequencing techniques. For example, putative driver mutations were identified that are unique to a subset of the clones of an individual bladder tumor detected through single cell sequencing [48]. We expect more detailed studies in the future to test the patterns that we have observed in the meta-analysis presented here.

## Conclusions

In a meta-analysis of genome variation data from multiple tumor in each patient, we find that the numbers of late driver mutations are substantial: they often exceed the number of early drivers. No previous study has conclusively demonstrated this pattern, even though they have indicated presence of driver mutations in tumors. These results implicate driver mutations in the continued development of aggressive tumor growth and in progression during later events such as recurrence, metastasis well beyond the initial founding of the tumor. Finally, these results highlight the importance of accounting for intratumor heterogeneity when evaluating the mutational histories of tumor cell populations.

## Additional file

Additional file 1: Our designations of drivers using five annotation schemes, chromosomal positions, count of samples with mutant allele after Treeomics treatment, patient ID, and study information are shown. (XLSX 2387 kb)

## Abbreviations

CCF: Cancer cell fraction
Indel: Insertions and deletions
M-seq: Multi-region sequencing
SNV: Single nucleotide variants
TCGA: The cancer genome atlas
